# Divergence-Based Introgression Polarization

**DOI:** 10.1093/gbe/evaa053

**Published:** 2020-03-27

**Authors:** Evan S Forsythe, Daniel B Sloan, Mark A Beilstein

**Affiliations:** e1 Department of Biology, Colorado State University; e2 School of Plant Sciences, University of Arizona

**Keywords:** introgression, hybridization, phylogenomics, phylogenetics

## Abstract

Introgressive hybridization results in the transfer of genetic material between species, often with fitness implications for the recipient species. The development of statistical methods for detecting the signatures of historical introgression in whole-genome data has been a major area of focus. Although existing techniques are able to identify the taxa that exchanged genes during introgression using a four-taxon system, most methods do not explicitly distinguish which taxon served as donor and which as recipient during introgression (i.e., polarization of introgression directionality). Existing methods that do polarize introgression are often only able to do so when there is a fifth taxon available and that taxon is sister to one of the taxa involved in introgression. Here, we present divergence-based introgression polarization (DIP), a method for polarizing introgression using patterns of sequence divergence across whole genomes, which operates in a four-taxon context. Thus, DIP can be applied to infer the directionality of introgression when additional taxa are not available. We use simulations to show that DIP can polarize introgression and identify potential sources of bias in the assignment of directionality, and we apply DIP to a well-described hominin introgression event.

## Introduction

Hybridization is an influential evolutionary force (Stebbins 1969) that is widespread in natural populations (Yakimowski and Rieseberg 2014; Mallet et al. 2016). Through backcrossing to parental populations, hybrids can serve as bridges for the transfer of alleles and adaptive traits between species or populations, a process known as introgression (Rieseberg and Soltis 1991; Rieseberg et al. 1996; Green et al. 2010; Dasmahapatra et al. 2012; Mallet et al. 2016; Suarez-Gonzalez et al. 2016). Whole-genome sequences and advances in phylogenetic methods (Soltis and Soltis 2003) have revealed signatures of historical introgression in scientifically and economically important groups, including well-studied examples in Neanderthals and non-African human populations (Green et al. 2010; Kuhlwilm et al. 2016). Several methods have been developed to identify taxa that exchanged genes during introgression (Huson et al. 2005; Than et al. 2008; Green et al. 2010; Durand et al. 2011; Liu et al. 2014; Martin et al. 2015; Pease and Hahn 2015; Stenz et al. 2015; Rosenzweig et al. 2016). Although these methods generally perform well across a variety of biological and experimental scenarios (Zheng and Janke 2018), theoretical and empirical studies have identified conditions under which each method is susceptible to bias (Eriksson and Manica 2012; Rosenzweig et al. 2016).

One challenging aspect of analyzing introgression is to identify taxa serving as donors versus recipients of genetic material during introgression (i.e., introgression directionality). If hybrids successfully backcross to both parents, alleles will move in both directions, meaning each parent will serve as donor for some introgressed loci and recipient for other loci. However, if backcrosses with one parent but not the other are favored by physiological (Rieseberg and Soltis 1991), selective (Orive and Barton 2002), or biogeographical (Currat et al. 2008) factors, it can lead to asymmetrical (Barton and Hewitt 1985) movement of alleles (directional introgression, denoted hereafter with ⇒). Introgression has been shown to underlie the transfer of adaptive traits to recipient lineages (Whitney et al. 2006; Dasmahapatra et al. 2012; Dannemann et al. 2016; Figueiró et al. 2017), so the ability to infer the directionality of introgression (i.e., polarize introgression) is essential in order to form hypotheses about functional and adaptive consequences.

The majority of tests to detect the occurrence of introgression do not explicitly polarize directionality (Zheng and Janke 2018), and those that can only do so in certain cases. For example, the *D*-statistic (Green et al. 2010) is widely used to infer instances of introgression in a four-taxon system. Introgression polarization is possible under *D* only when data for a fifth taxon are available (Green et al. 2010; Eaton and Ree 2013; Eaton et al. 2015; Pease and Hahn 2015). Moreover, the fifth taxon must be sister to one taxon involved in introgression but cannot itself be involved in introgression. (Pease and Hahn 2015) define this specific configuration of introgressing taxa and sister taxa as “intergroup” introgression and describe how, when these specific five-taxon conditions are met, the branching order of introgressed gene trees indicates directionality. However, the authors also describe how other types of introgression (e.g., “ancestral” introgression) cannot be polarized. Moreover, there are many cases in which a fifth taxon with the required phylogenetic placement is either not sampled or does not exist. In these cases, it is possible to statistically identify introgression using existing methods but not necessarily to polarize introgression. Thus, there is a need for a more widely applicable statistical method to distinguish between bidirectional and unidirectional introgression, while identifying donor and recipient taxa.

**Fig. 1.**
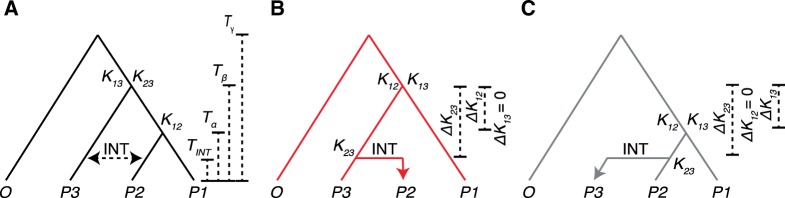
—Expected divergence under simulated introgression. The species *P1*, *P2*, *P3*, and *O* were used for simulation analyses. (*A*) The species branching order. Introgression between species *P2* and *P3* is indicated with a double-sided dotted arrow. Default values used during all simulations, unless specified otherwise, are: *T*_INT_=1, *T*_α_*=*4, *T*_β_=8, and *T*_γ_=12 in coalescent units (4*N* generations) (Hudson 2002). (*B*) A gene tree depicting a gene that was introgressed *P3⇒P2*. (*C*) A gene tree depicting a gene that was introgressed *P2⇒P3*. Δ*K* values are calculated based on changes in mean divergence between pairs of taxa in the set of trees with the speciation topology versus the set of introgression trees (see eqs 1–3). Note that the expected profiles of Δ*K* values for *P3⇒P2* introgression differs from that of *P2⇒P3* introgression, forming the basis for the DIP test (see main text and fig. 2).

Here, we describe and test a method for inferring directionality of introgression from genome-scale data, which we refer to as divergence-based introgression polarization (DIP). DIP is based on the observation that, when introgression occurs, it alters not only the level of nucleotide sequence divergence between the two species exchanging genes (Rosenzweig et al. 2016) but also divergences with related species that are not directly involved in introgression; these changes occur in systematic and predictable ways according to the directionality of introgression (fig. 1) (Forsythe et al. 2018; Fontaine et al. 2015; Hibbins and Hahn 2019). DIP is calculated from pairwise sequence divergence between taxa involved in introgression and a sister taxon, comparing divergence values obtained from introgressed loci versus nonintrogressed loci. It takes as input the same types of data used to infer introgression by existing methods (whole-genome/chromosome alignments or single-gene alignments of loci sampled throughout the genome). However, unlike most existing methods, DIP is applicable to cases in which only four taxa are sampled, thereby expanding inference of introgression directionality to a broader scope of evolutionary histories.

We present tools to implement the DIP method: https://github.com/EvanForsythe/DIP. We also simulate whole-genome alignments in which a subset of loci was introgressed either unidirectionally, asymmetrically, or symmetrically. We use these simulated genome alignments to assess how accurately DIP polarizes asymmetrical introgression and to investigate the effects of parameters that are known to affect existing introgression inference methods, such as the proportion and timing of introgression (Durand et al. 2011; Martin et al. 2015; Zheng and Janke 2018). We have recently used the principles of DIP to document asymmetrical introgression among Brassicaceae species (Forsythe et al. 2018), and here, we also apply DIP to empirical data from modern and archaic hominins.

## New Approaches

Introgression alters levels of sequence divergence between taxa, and these changes can differ depending on directionality (Forsythe et al. 2018; Hibbins and Hahn 2019) (fig. 1). Although several statistics focus on the effects of introgression on sequence divergence between species involved in introgression (Feder et al. 2005; Joly et al. 2009; Rosenzweig et al. 2016), here, we describe how patterns of sequence divergence in a taxon that is sister to those involved in introgression can be indicative of the directionality of introgression. To define the properties of a divergence-based introgression test, we use hypothetical species *P1*, *P2*, *P3* and an outgroup, *O*. Species *P1* and *P2* are sister within the species tree, and we model introgression between species *P2* and *P3*. We denote the timing of the three successive speciation events among these taxa as *T*_γ_, *T*_β_, and *T*_α_ and the timing of the introgression event between *P2* and *P3* as *T*_INT_ (fig. 1*A*). When introgression has occurred between *P2* and *P3*, some loci will reflect a history of introgression, whereas other loci will reflect a history of speciation. In applying DIP, a gene tree is inferred for each locus, and the resulting topology is used to distinguish introgressed loci from nonintrogressed loci. For all loci, we quantify pairwise sequence divergence values between *P2* and *P3* (*K*_23_), between *P1* and *P2* (*K*_12_), and between *P1* and *P3* (*K*_13_) (fig. 1). The values of *K*_23_, *K*_12_, and *K*_13_ on a given gene tree are expected to correspond to *T*_INT_, *T*_α_, and *T*_β_ in a way that depends on the introgression history of that gene. Note that *K*_23_ is the divergence measurement that is most commonly used to indicate the presence of introgression (Feder et al. 2005; Joly et al. 2009; Rosenzweig et al. 2016) because introgression in either direction is expected to reduce *K*_23_ relative to genes that reflect the species tree, as the divergence time between the sequences of these taxa is reduced from *T*_β_ to *T*_INT_ (fig. 1). In contrast, changes in *K*_12_ and *K*_13_ will depend on the direction of introgression. For example, introgression can cause *K*_12_ to increase corresponding to a change in divergence time from *T*_α_ to *T*_β_ but only if introgression occurred from *P3* to *P2* (fig. 1*B*). Introgression in the other direction should not affect *K*_12_. The effects on *K*_13_ are also sensitive to the direction of introgression. If it occurs from *P2* to *P3*, introgression should decrease *K*_13_ based on a change in divergence time from *T*_β_ to *T*_α_ (fig. 1*C*), but there should be no effect on *K*_13_ if introgression occurs in the other direction. To quantify these effects, differences are calculated between the mean values of *K*_23_, *K*_12_, and *K*_13_ from all loci displaying the species topology (abbreviated SP loci in equations/figures) and the mean values of the same corresponding divergence measurements from all loci displaying the introgression topology (abbreviated INT loci in equations/figures) in the following fashion:

(1) $$\Delta K_{23}\;=\;{\bar{K}}_{23}\left(\mathrm{SP}\;\mathrm{loci}\right)\;-\;{\bar{K}}_{23}\left(\mathrm{INT}\;\mathrm{loci}\right)$$

(2) $$\Delta K_{12}\;=\;{\bar{K}}_{12}\left(\mathrm{INT}\;\mathrm{loci}\right)\;-\;{\bar{K}}_{12}\left(\mathrm{SP}\;\mathrm{loci}\right)$$

(3) $$\Delta K_{13\;}=\;{\bar{K}}_{13}\left(\mathrm{SP}\;\mathrm{loci}\right)\;-\;{\bar{K}}_{13}\left(\mathrm{INT}\;\mathrm{loci}\right)$$

Note that the order of subtraction used in defining these terms is not always the same with respect to species and introgression loci and was chosen such that the effects of relevant introgression are expected to yield positive (rather than negative) Δ*K* in each case. Together, this set of Δ*K* values composes the divergence profile of DIP. Below, we show the relative magnitudes of these values can be used to differentiate evolutionary histories based on the polarity of introgression. We also use coalescent-based simulations to identify biases that can be introduced by other sources of genealogical discordance such as incomplete lineage sorting (ILS), and we devise additional layers of DIP comparisons that can be used to partially alleviate these biases.

## Results

### DIP: Distinguishing Modes of Unidirectional and Bidirectional Introgression

**Fig. 2.**
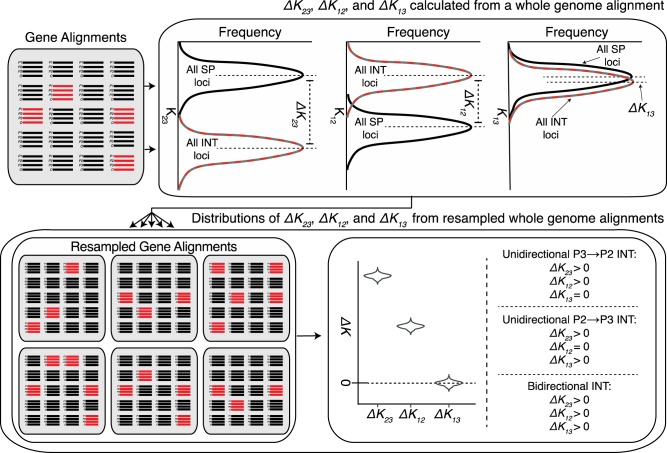
—Workflow of the DIP test. Point estimates of Δ*K*_23_, Δ*K*_12_, Δ*K*_13_ are calculated from whole genomes, which are then resampled to yield distributions of Δ*K*_23_, Δ*K*_12_, Δ*K*_13._ Unidirectional *P3⇒P2* introgression is indicated by the profile, Δ*K*_23_ > 0, Δ*K*_12_ > 0, and Δ*K*_13_ = 0. Unidirectional *P2⇒P3* introgression is indicated by Δ*K*_23_ > 0, Δ*K*_12_ = 0, and Δ*K*_13_ > 0. Bidirectional introgression is indicated by Δ*K*_23_ > 0, Δ*K*_12_ > 0, and Δ*K*_13_ > 0. All other profiles are considered inconclusive regarding the occurrence and directionality of introgression. *P* values for testing whether each Δ*K* value significantly differs from 0 are obtained from the proportion of replicates for which Δ*K* ≤ 0. Colors reflect the black, red, and gray genealogical histories from figure 1. In this illustration, all introgression loci are in the *P3⇒P2* (red) direction. However, we use the red/gray dashed lines for showing the distribution of introgression loci because, in general, the set of introgression loci can contain *P3⇒P2* loci, *P2⇒P3* loci, or both.

**Fig. 3.**
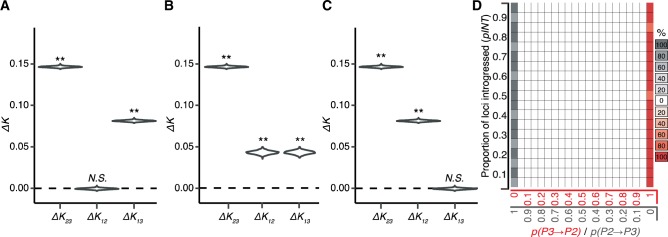
—DIP analysis of simulated introgression. Genomes were simulated according to steps 1–3 in supplementary figure S1, Supplementary Material online, under unidirectional *P2⇒P3* introgression (*A*), symmetrical bidirectional *P3*⇔*P2* introgression (*B*), and unidirectional *P3⇒P2* introgression (*C*). Simulation parameters are as follows: (*A*), *n* = 5,000, *pINT* = 0.5, *p(P3⇒P2)* = 0; (*B*), *n* = 5,000, *pINT* = 0.5, *p(P3⇒P2)* = 0.5; (*C*), *n* = 5,000, *pINT* = 0.5, *p(P3⇒P2)* = 1. DIP was applied to each genome to yield profiles of Δ*K*_23_, Δ*K*_12_, Δ*K*_13_*.* ** indicates significant departure from 0 (*P* < 0.01). (*D*) A plot scanning simulation parameters, proportion of the genome that was introgressed (*pINT*) (*y* axes) and proportion of introgressed loci transferred in each direction (*p(P3⇒P2)*) (*x* axis). Each square in the plot indicates the DIP results obtained from five replicated simulated genome alignments. Red boxes indicate the profile consistent with *P3⇒P2* introgression (see panel *C*). Gray boxes indicate the profile consistent with *P2⇒P3* introgression (see panel *A*). The shading of the boxes corresponds the percentage of replicates that indicate a given profile, as specified by the key to the right of the plot. Unshaded boxes indicate zero replicates yielded a significant unidirectional profile (i.e., all replicates yield the bidirectional introgression profile; see panel *B*).

The simplest application of DIP involves testing whether Δ*K*_23_, Δ*K*_12_, and Δ*K*_13_ are significantly >0 and compares these results to the expectations for Δ*K* under different introgression scenarios (fig. 2). If introgression has occurred in both directions between *P2* and *P3*, then all three Δ*K* values should be positive. However, as noted above, if introgression has occurred exclusively in one direction, the expectation for either Δ*K*_12_ or Δ*K*_13_ should remain zero (fig. 2). To test the performance of DIP, we simulated alignments for thousands of loci (5,000 bp each) undergoing unidirectional introgression in each direction, as well as symmetric bidirectional introgression (see Materials and Methods and supplementary fig. S1, Supplementary Material online). We applied DIP to each simulated genome. For the genome simulated under unidirectional *P2⇒P3* introgression, we observed Δ*K*_23_ > 0, Δ*K*_12_ = 0, and Δ*K*_13_ > 0 (fig. 3*A*), which is the expected pattern for that direction of introgression (fig. 1). For the genome simulated under symmetric bidirectional introgression, we observed Δ*K*_23_ > 0, Δ*K*_12_ > 0, and Δ*K*_13_ > 0 (fig. 3*B*), which is the expected pattern if some introgression is occurring in both directions. For the genome simulated under unidirectional *P3⇒P2* introgression, we observed Δ*K*_23_ > 0, Δ*K*_12_ > 0, and Δ*K*_13_ = 0 (fig. 3*C*), again reflecting our expected DIP profile for that direction. These results indicate that DIP can correctly classify all three types of introgression under these simulated conditions.

Next, we explored the performance of DIP across a range of different parameter settings, including the proportions of genes in the genome that had been subject to introgression (*pINT*). We also varied the proportions of introgressed loci that moved in one direction or the other [*p(P3⇒P2)*]. We performed a parameter scan (supplementary fig. S1, Supplementary Material online) by generating simulated genomes with different values of *pINT* and *p(P3⇒P2)* and applying DIP to each genome (fig. 3*D*). We found the expected *P3⇒P2* DIP profile for the majority of replicated genomes generated with *p(P3⇒P2)*=1 (i.e., unidirectional *P3⇒P2* introgression) (fig. 3*D*, red boxes). Further, we found the expected *P2⇒P3* DIP profile for the majority of replicated genomes generated with *p(P3⇒P2)*=0 (i.e., unidirectional *P2⇒P3* introgression) (fig. 3*D*, gray boxes). Intermediate *p(P3⇒P2)* values all yielded the expected DIP profile for bidirectional introgression for all replicates (fig. 3*D*, white boxes). These simulations constitute the basic implementation of DIP (hereafter, referred to as single-DIP or 1×DIP), which can detect the presence of bidirectional introgression (see fig. 3*B* profile and fig. 3*D* white boxes), but does not report directional asymmetry (i.e., whether either of the two directions predominates) at intermediate values of *p(P3⇒P2)*.

### Double-DIP: Detecting Asymmetry in Cases of Bidirectional Introgression

Existing introgression polarization methods tend to assume unidirectionality of introgression, but it is also important to consider the possibility of asymmetric bidirectional introgression that falls short of being strictly unidirectional (discussed in Martin et al. 2015). To more directly test for asymmetry in cases of bidirectional introgression, we developed an additional step in the DIP analysis, which we refer to as double-DIP or 2×DIP. The premise of 2×DIP is that Δ*K*_12_ for loci introgressed *P3⇒P2* and Δ*K*_13_ for loci introgressed *P2⇒P3* have the same expected values, as they are both based on a shift in divergence time between *T*_β_ and *T*_α_ (fig. 1). Therefore, under symmetric bidirectional (*P3*⇔*P2*) introgression, we expect genome-wide values of Δ*K*_12_ and Δ*K*_13_ to equal each other. Alternatively, if *P3⇒P2* introgression exceeds *P2⇒P3* introgression, we expect genome-wide Δ*K*_12_ > Δ*K*_13_. *2*×DIP compares the magnitudes of Δ*K*_12_ and Δ*K*_13_ by formulating a simple summary statistic, ΔΔ*K*, which is defined as follows:

(4) $$\Delta \Delta K=\;\Delta K_{12}\;\;-\;\;\Delta K_{13}$$

**Fig. 4.**
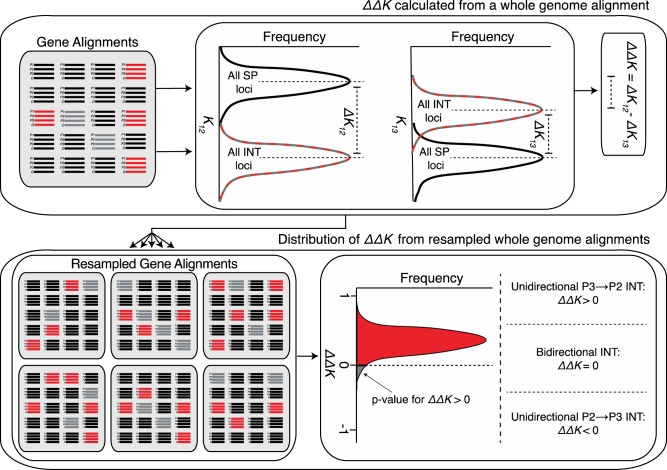
—Workflow of the 2×DIP test. (Top) A point estimate of ΔΔ*K* is calculated from a whole-genome alignment from Δ*K*_12_ and Δ*K*_13_ values. (Bottom) A sampling distribution of ΔΔ*K* is calculated from resampled gene alignments (bootstrapping) obtained from the original genome. If the majority of ΔΔ*K* replicates are > 0, it is an indication of asymmetric *P3⇒P2* introgression. In this case, the proportion of ΔΔ*K* replicates <0 determines the *P* value (doubled for a two-sided test) for asymmetric *P3⇒P2* introgression. Asymmetric *P2⇒P3* introgression is indicated by the opposite pattern.

The expectation for the ΔΔ*K* summary statistic is zero under symmetric bidirectional introgression, positive under introgression that is biased toward *P2*, and negative under introgression that is biased toward *P3* (fig. 4).

**Fig. 5.**
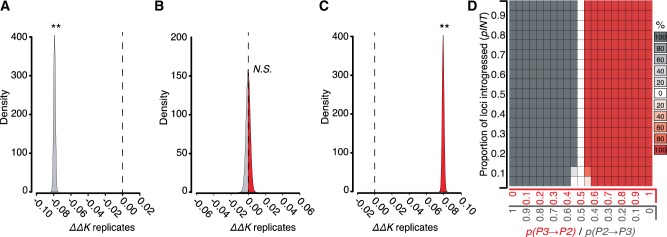
—2×DIP analysis of simulated introgression. Genomes were simulated according to steps 1–3 in supplementary figure S1, Supplementary Material online. Genomes were simulated under unidirectional *P2⇒P3* introgression (*A*), symmetrical bidirectional *P3*⇔*P2* introgression (*B*), and unidirectional *P3⇒P2* introgression (*C*). Simulation parameters are as follows: (*A*), *n* = 5,000, *pINT* = 0.5, *p(P3⇒P2)* = 0; (*B*), *n* = 5,000, *pINT* = 0.5, *p(P3⇒P2)* = 0.5; (*C*), *n* = 5,000, *pINT* = 0.5, *p(P3⇒P2)* = 1. 2×DIP was applied to each genome to yield a sampling distribution of ΔΔ*K*. ** indicates significant departure from 0 (*P* < 0.01). (*D*) A plot scanning *pINT* and *p(P3⇒P2)* as in figure 3*D*. Red boxes indicate significant (*P*<0.05) *P3⇒P2* 2×DIP signature (see panel *C*). Gray boxes indicate significant (*P*<0.05) *P2⇒P3* 2×DIP signature (see panel *A*). Five replicate genomes were simulated for each parameter value. The shading of the boxes corresponds the percentage of replicates for which 2×DIP significantly indicated a directional signature, as specified by the key to the right of the plot. Unshaded boxes indicate zero replicates yielded a significant directional signature (i.e., all five replicates failed to reject the null hypothesis of symmetrical introgression; see panel *B*).

We explored the performance of 2×DIP by simulating genomes in the same manner as described above for 1×DIP. For the genome simulated under unidirectional *P2⇒P3* introgression (*p(P3⇒P2)* = 0), we observed a significantly negative ΔΔ*K* (fig. 5*A*, *P < *0.0002), consistent with our expectations. For the genome simulated under symmetric bidirectional introgression, ΔΔ*K* did not significantly differ from zero (fig. 5*B*, *P = *0.914), also consistent with expectations. For the genome simulated under unidirectional *P3⇒P2* introgression (*p(P3⇒P2)* = 1), we observed significantly positive ΔΔ*K* (fig. 5*C*, *P *<* *0.0002), again reflecting expectations. These results indicate that 2×DIP correctly classified all three types of simulated introgression events. As above, we also performed a parameter scan to explore 2×DIP. We found that genomes simulated with *p(P3⇒P2)* = 0.5 (i.e., symmetric bidirectional introgression) returned ΔΔ*K* value that did not significantly differ from zero (fig. 5*D*, white boxes). We also found significant ΔΔ*K <* 0 for nearly all replicated genomes simulated with *p(P3⇒P2)* < 0.5 and significant ΔΔ*K >* 0 for nearly all replicated genomes simulated with *p(P3⇒P2)* > 0.5 (fig. 5*D*). The only exception to these patterns was found when 10% or less of loci in the simulated genome (*pINT* ≤ 0.1) underwent nearly symmetrical introgression (*p(P3⇒P2)* = 0.45 and 0.55).

To test the influence of recombination on DIP performance, we also applied an alternative simulation approach in which full chromosomes were simulated under different rates of recombination (resulting in varying haplotype block sizes), while applying the same 5,000-bp partition size used in our other analyses (see Materials and Methods). We found that 2×DIP correctly inferred unidirectional introgression regardless of recombination rate (supplementary fig. S2, Supplementary Material online; *p(P3⇒P2)* = 0 and 1) and reliably detected slight (*p(P3⇒P2)* = 0.4 and 0.6) directional asymmetries when the size of haplotype blocks was the same or smaller than the size of the sliding window applied during DIP (supplementary fig. S2*B* and *C*, Supplementary Material online). However, when haplotype blocks were an order of magnitude larger than the window size, we observed increased noise in DIP at intermediate *p(P3⇒P2)* values (supplementary fig. S2*A*, Supplementary Material online), likely due to pseudoreplication caused by many trees reflecting the exact same genealogy (supplementary fig. S2*D*, Supplementary Material online), ultimately leading to increased sampling variance (see Discussion). Taken together, these results indicate that 2×DIP correctly inferred asymmetrical introgression, even in many cases in which there is only slight asymmetry, meaning it is a sensitive method for polarizing asymmetrical introgression that is robust across a variety of parameter values.

### Robustness of DIP to Population Divergence Time

The task of accurately classifying loci as introgressed versus nonintrogressed (i.e., INT loci vs. SP loci, respectively) based on gene tree topology is an integral part of DIP; however, this task is confounded when the topology of a gene tree does not accurately reflect the history of introgression (or lack thereof) that occurred at that locus. For example, phylogenetic methods rely on diagnostic synapomorphies to infer gene tree topologies; scarcity of synapomorphies or large amounts of homoplasy in an alignment can lead to phylogenetic error and, thus, inaccurate classification. Another important confounding factor is ILS, which can result in gene trees that reflect a history of deep coalescence at a locus, as opposed to the underlying history of speciation and/or introgression at that locus. This process can result in nonintrogressed loci displaying the introgressed topology. Alternatively, because ILS and introgression are not mutually exclusive processes, ILS can also lead to introgressed loci displaying the species topology. Importantly, ILS is also expected to yield gene trees displaying an alternative third topology that is neither the species topology or the introgressed topology (Green et al. 2010) (see Triple-DIP below).

Both phylogenetic error and ILS are more pronounced during rapid divergence (i.e., short internal branches) (Fontaine et al. 2015). Moreover, it has been shown that, because *P3⇒P2* introgression trees have longer internal branch lengths than *P2⇒P3* introgression trees, the latter are more prone to both phylogenetic error and ILS (Zheng and Janke 2018), ultimately leading them to be more prone to misclassification in DIP. This feature introduces the potential for directional bias in DIP (see Discussion). Therefore, we explored divergence times, as an additional parameter that may influence performance. We focus our discussion on the process of ILS, but it should be noted that phylogenetic error also has the potential to occur in empirical data sets.

**Fig. 6.**
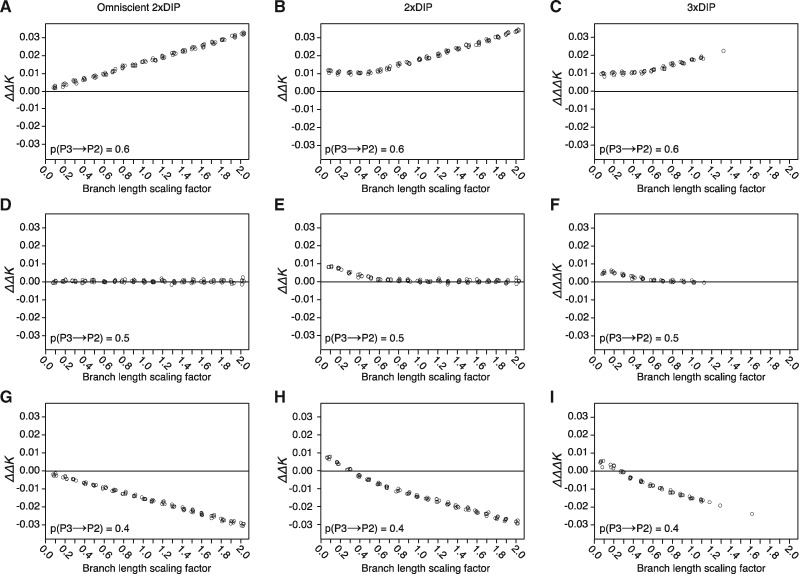
—Exploration of branch length parameters used during genome simulation. The default branch lengths used during all previous simulations (*T*_IG_=1, *T*_α_=4, T_β_=8, and *T*_γ_=12) were multiplied by branch-length scaling factors. For all plots, five replicate genomes were simulated for each scaling factor value. *pINT* = 0.5 was used for all simulations. DIP was performed on each replicate; individual points on plots represent point estimates of ΔΔ*K* and ΔΔΔ*K* (jittered for clarity). Genomes were simulated with asymmetric introgression favoring *P3⇒P2* (*A*–*C*), symmetric bidirectional introgression (*D*–*F*), and asymmetric introgression favoring *P2⇒P3* (*G*–*I*). Omniscient 2×DIP (*A*, *D*, and *G*), standard 2×DIP (*B*, *E*, and *H*), and 3×DIP (*C*, *F*, and *I*) were performed. ΔΔΔ*K* data points are absent at higher scaling factors because this adjusted version of ΔΔ*K* can only be calculated when there are at least some loci with the unexpected topology (ALT loci) as a result of topology misclassification or ILS.

All previous simulations were implemented with constant and large divergence times (see fig. 1). To explore the branch length parameter, we modified divergence times by multiplying all of the branch lengths by a scaling factor (SF) (see Materials and Methods), essentially modifying the height of the entire tree used for simulations. SFs >1 yield taller trees, whereas SFs <1 yield shorter trees. For each SF, we simulated five replicate genomes and calculated ΔΔ*K* for each replicate. We first classified introgressed and nonintrogressed loci based on the known history used to simulate the data and plotted the resulting ΔΔ*K* values (omniscient 2×DIP). We found that 2×DIP correctly inferred asymmetry (or lack thereof) at all branch lengths and that the magnitude of ΔΔ*K* was proportional to the SF (fig. 6*A*, *D*, and *G*). However, when working with real data sets it is rare to know if individual loci with introgression topologies are the result of bona fide introgression, as opposed to ILS or errors in phylogenetic inference. To explore the impact of the SF on the ability of 2×DIP to distinguish between a signature of bona fide introgression versus the effects of ILS, we calculated ΔΔ*K* using topology-based (non-omniscient) classification. With this approach, we observed an upward bias in ΔΔ*K* at low SFs (fig. 6*B*, *E*, and *H*). This bias favors inference of *P3⇒P2* introgression even when there is asymmetry in the opposite direction (fig. 6*E*). As expected, this bias exists at the SFs for which misclassification of gene trees is most pronounced (supplementary fig. S3, Supplementary Material online), suggesting that it results from ILS (see Discussion).

We also explored the influence of the timing of introgression relative to speciation nodes. We held the timing of speciation constant while varying only the timing of the introgression event (i.e., relative introgression time). We found that 2×DIP accurately polarizes asymmetric introgression in all cases under omniscience (supplementary fig. S4*A* and *D*, Supplementary Material online). Under non-omniscience, 2×DIP is accurate when speciation and introgression are separated by a substantial period of time (i.e., relatively recent introgression times) (supplementary fig. S4*B*, Supplementary Material online). However, we observe a bias in favor of inference of *P3⇒P2* introgression (similar to the bias described above) when introgression occurs immediately following speciation (supplementary fig. S4*B*, Supplementary Material online) and this effect is compounded when total tree-height is small (i.e., SF = 0.1) (supplementary fig. S4*E*, Supplementary Material online). Below, we explore sources of bias and strategies for mitigating its effects.

### Triple-DIP: Adjusting for Gene Tree Classification Bias

To address the directional bias in 2×DIP caused by gene tree ILS at short branch lengths, we developed an additional layer that can be applied in DIP analysis, which we refer to as triple-DIP or 3×DIP, so named because it includes an additional Δ component (i.e., the “delta of the delta of the delta”). Briefly, in addition to calculating the standard 2×DIP as above, we also calculate an alternative ΔΔ*K* (ΔΔ*K*_ALT_) that substitutes gene trees with the alternative topology, ((*P1*, *P3*), *P2*), for the introgressed loci used in the standard ΔΔ*K*:

(5) $$\Delta \Delta K_{\mathrm{ALT}}=\left({\bar{K}}_{12}\left(\mathrm{ALT}\;\mathrm{loci}\right)\;-\;{\bar{K}}_{12}\left(\mathrm{SP}\;\mathrm{loci}\right)\right)\;-\;\;\left({\bar{K}}_{23}\left(\mathrm{SP}\;\mathrm{loci}\right)\;-\;{\bar{K}}_{23}\left(\mathrm{ALT}\;\mathrm{loci}\right)\right)$$

Note that, *K*_23_ values are substituted in place of *K*_13_ values in calculating this version of ΔΔ*K* because we are now focusing on a conflicting topology in which *P1* and *P3* are sister to each other. Because *P2* and *P3* are the two taxa subject to introgression, loci with this alternative topology should arise only from ILS and not introgression. Following the logic of standard *D*-statistics (Green et al. 2010; Durand et al. 2011), we reasoned that ILS should be equally likely to produce each of the two topologies that conflict with the species tree. Therefore, this alternative 2×DIP calculation may provide a measure of the amount of bias that is introduced by ILS. In applying 3×DIP, we weight this value by the counts of loci with the expected (*P3*⇔*P2*) introgression topology (*N*_INT_ loci) and the alternative topology (*N*_ALT_ loci). The ΔΔΔ*K* summary statistic is calculated as follows (see Materials and Methods for derivation):

(6) $$\Delta \Delta \Delta K=\frac{\left(\Delta \Delta K\;\;\times \;\;N_{\mathrm{INT}}\right)\;-\;(\Delta \Delta K_{\mathrm{ALT}}\;\;\times \;\;N_{\mathrm{ALT}})\;}{N_{\mathrm{INT}}\;-\;N_{\mathrm{ALT}}}$$

It should be noted that calculation of a 3×DIP correction is only possible when there is at least some ILS because it relies on the presence of ((*P1*, *P3*), *P2*) loci. As such, when we applied 3×DIP to genomes simulated with different branch lengths, we were only able to consistently obtain measurements under short-branch conditions (SF < 1.0) where ILS is prevalent (fig. 6*C*, *F*, and *I*) because these were the only conditions that returned some loci with the relevant topology. Under these short-branch conditions, we found that 3×DIP reduced but did not eliminate the bias observed in 2×DIP. Although ΔΔΔ*K* was still erroneously positive for the lowest branch length values (fig. 6*F* and *I*), the magnitude of ΔΔΔ*K* was less than that of ΔΔ*K.*

**Fig. 7.**
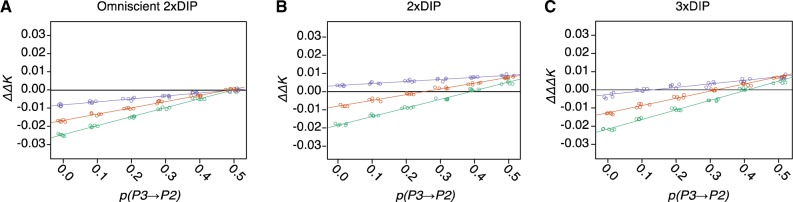
—Characterization of DIP bias under short branch conditions. Genomes were simulated with different values of *p(P3⇒P2)* (*x* axis) and different branch-length scaling factors (SF) (point colors). See figure 6 for description of SF. Purple, SF = 0.1; Orange, SF = 0.2; Green, SF = 0.3. As in figure 6, Omniscient 2×DIP (*A*), standard 2×DIP (*B*), and 3×DIP (*C*) were performed. Five replicate genomes were analyzed for each condition. *pINT* = 0.5 was used for all simulations.

We further explored bias in 2×DIP and 3×DIP by simulating short branch trees (with SF of 0.1, 0.2, and 0.3) across a range of *p(P3⇒P2)* values. We first applied omniscient 2×DIP to give context to the bias introduced. As expected, omniscient 2×DIP yielded negative ΔΔ*K* values for all replicates in which *p(P3⇒P2)* < 0.5 (fig. 7*A*). Consistent with the bias observed in figure 6, standard (non-omniscient) 2×DIP yielded erroneously positive ΔΔ*K* values, especially for the shortest branch length conditions (fig. 7*B*). 3×DIP reduced the bias, only yielding erroneously positive ΔΔΔ*K* values for the highest *p(P3⇒P2)* values and the shortest branch length conditions (fig. 7*C*). We also tested the performance of DIP in a situation in which ILS has occurred but not introgression (*pINT *=* *0; SF = 0.1) (supplementary fig. S5, Supplementary Material online). Despite the lack of true introgression in these simulations, 1×DIP produced a profile consistent with *P3⇒P2* introgression (supplementary fig. S5*B*, Supplementary Material online), although the relative positions of Δ*K*_23_, Δ*K*_12_, and Δ*K*_13_ distributions differed from the pattern in figure 3*C*. 2×DIP also significantly indicated *P3⇒P2* introgression (supplementary fig. S5*C*, Supplementary Material online), but 3×DIP produced a ΔΔΔ*K* that was not significantly different from zero, again indicating that 3×DIP is less prone to falsely indicating *P3⇒P2* introgression. However, when we explored bias in the context of relative introgression timing (as opposed to total tree-height), we found some situations in which 3×DIP showed increased directional bias compared with 2×DIP (supplementary fig. S4, Supplementary Material online). 3×DIP bias exceeded 2×DIP bias in situations in which total tree-height was large (high SFs) (supplementary fig. S4*G*, Supplementary Material online) but the opposite was true for low SFs (supplementary fig. S4*H*, Supplementary Material online). Together, these results indicate that 3×DIP reduces bias in some (but not all) situations, meaning that information can be gained by applying both 2×DIP and 3×DIP when analyzing empirical data.

### Analysis of Hominin Introgression

**Fig. 8.**
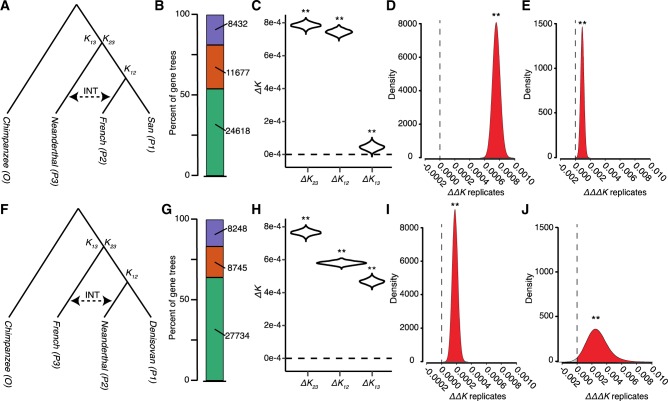
—DIP analysis of hominin introgression. DIP was performed on whole-chromosome alignments of chromosome 1 using two different taxon-sampling schemes (TSS). (*A*) Depiction of the samples used in TSS1. (*B*) Neighbor-joining gene-tree topologies from individual loci. (San.,French),Nean.), green; (French, Nean.),San), orange; (San, Nean.),French), purple. (*C*–*E*) Results from 1×DIP (*C*), 2×DIP (*D*), and 3×DIP (*E*) applied to TSS1 alignment. (*F*) Depiction of the sampled used in TSS2. (*G*) Neighbor-joining gene-tree topologies from individual loci. (Deni.,Nean.),French), green; (Nean.,French),Deni.), orange; (Deni.,French),Nean.), purple. (*H*–*J*) Results from 1×DIP (*H*), 2×DIP (*I*), and 3×DIP (*J*) applied to TSS2 alignment. ** indicates significant departure from 0 (*P *<* *0.01).

To understand the performance of DIP on empirical data, we applied DIP to existing genomic data for introgression that occurred between Neanderthal and a modern human European lineage (Green et al. 2010; Prüfer et al. 2014). Applying a five-taxon version of the *D-*statistic that made use of the phylogenetic position of multiple modern African populations, a previous study (Green et al. 2010) determined that unidirectional introgression occurred from Neanderthal to European lineages. We applied DIP to Chromosome 1 from a Neanderthal sample, a Denisovan sample, two modern human (San [African] and French [European]) samples, and the chimpanzee reference genome. The availability of a Denisovan sample allowed us to infer DIP in two different ways using two different taxon-sampling schemes (TSS1 and TSS2) (fig. 8*A* and *F*). For both TSSs, there were three gene tree topologies present (fig. 8*B* and *G*), indicating the possibility of misclassification due to phylogenetic error and ILS.

Using TSS1, 1×DIP yielded a profile indicating the presence of at least some bidirectional introgression (fig. 8*C*), a scenario which was not ruled out by Green et al. (2010). However, it should be noted that, whereas Δ*K*_12_ and Δ*K*_13_ were both significantly positive, the Δ*K*_13_ was much closer to zero, which would indicate a substantial asymmetry toward Neanderthal⇒French introgression. 2×DIP and 3×DIP indicated significantly positive ΔΔ*K* and ΔΔΔ*K*, respectively (fig. 8*D* and *E*), consistent with asymmetric introgression in the Neanderthal⇒French direction. However, when we applied DIP to TSS2, we saw contradictory results. While 1×DIP again indicated the presence of bidirectional introgression, although without the near-zero Δ*K*_13_ (fig. 8*H*), 2×DIP and 3×DIP yielded positive ΔΔ*K* and ΔΔΔ*K*, respectively (fig. 8*I* and *J*). 2×DIP and 3×DIP from TSS2 thus indicate French⇒Neanderthal introgression. Although introgression from modern humans has been inferred in other Neanderthal samples (Kuhlwilm et al. 2016), it is at odds with findings from TSS1 and Green et al. (2010).

To understand this discrepancy and put our empirical analyses in the context of our simulations, we plotted distributions of divergence estimates (*K*_23_, *K*_12_, and *K*_13_) calculated from two simulated genomes and the TSSs used for the empirical analysis (supplementary fig. S6, Supplementary Material online). The empirical distributions display a wider spread than the simulated distributions, potentially introducing noise into the empirical analysis. Importantly, empirical data also show reduced levels of divergence, even compared with the data set simulated with the shortest branch lengths (SF = 0.1). This suggests that the biasing factors explored above could be even more at play in the hominin analysis (see Discussion).

## Discussion

### Intended Applications of DIP

Our simulation analyses provide a proof-of-principle that divergence data can be used to polarize introgression in a four-taxon context, narrowing the methodological gap between our ability to identify introgression and our ability to determine the direction of gene transfer. It should be noted that DIP is not designed to replace existing methods and act as a frontline test of whether introgression has occurred. Instead, we recommend cases of introgression first be confidently identified with existing tools (Huson et al. 2005; Than et al. 2008; Green et al. 2010; Durand et al. 2011; Martin et al. 2015; Pease and Hahn 2015; Stenz et al. 2015; Rosenzweig et al. 2016). In these cases, DIP can then be used to polarize the direction of introgression, a critical step toward interpreting biological implications. As we have shown above, DIP has the potential to distinguish unidirectional and bidirectional introgression and, in cases of bidirectionality, to test for asymmetry between the two directions.

Although there are population genetic (Schrider et al. 2018) and five-taxon phylogenetic (Green et al. 2010; Pease and Hahn 2015) methods capable of polarizing introgression, DIP offers the ability to detect asymmetric introgression in both directions using a four-taxon context. This will be valuable because very little is known about the extent of reciprocal exchange that occurred during even well-studied introgression events (Green et al. 2010; Kuhlwilm et al. 2016), a deficit that likely stems from an absence of sensitive tools. Another group (Hibbins and Hahn 2019) has recently proposed an approach that overlaps with DIP. They introduce a statistic, *D*_2_, which is conceptually similar to Δ*K*_13_ described here. As such, nonzero values of *D*_2_ indicate the presence of *P2⇒P3* introgression (B⇒C by their nomenclature). DIP goes further than this approach because it also uses Δ*K*_12_ to test for introgression in the opposite direction and ΔΔ*K* to determine the predominant direction of introgression. The primary focus of the recent work by Hibbins and Hahn (2019) is the development of another statistic, *D*_1_, that assesses the timing of introgression relative to speciation events and can be used in assessing possible cases of homoploid hybrid speciation. This is an elegant application of the same type of divergence-based logic that underlies DIP to a biological question that cannot currently be addressed with our method. We suggest that further improvements in polarizing introgression can be made by combining the explicit coalescent-based modeling of Hibbins and Hahn (2019) with the more comprehensive summary provided by 1×, 2×, and 3×DIP.

### Bias in DIP

It should be noted that the simulation branch length parameters used in figures 3 and 5 resulted in gene trees with relatively deep divergences. These branch lengths were chosen because they emphasize differences in divergence and minimize potential biasing factors, thus providing the clearest view of the general properties of DIP. However, it has been shown that timing of population divergence is an extremely influential parameter in introgression analyses (Durand et al. 2011; Martin et al. 2015; Zheng and Janke 2018). This is true, in part, because the length of internal branches is directly related to the extent of ILS that occurs (Maddison and Knowles 2006). Short branches lead to increased ILS (Degnan and Rosenberg 2013), which can mimic introgression and introduce noise and bias into introgression analyses. Coalescent simulations, such as those that we performed, capture this phenomenon (Hudson 2002; Degnan and Rosenberg 2009), introducing discordant gene trees at a rate dependent on branch length parameters.

Population divergence is additionally important for DIP for a more intuitive reason; the magnitude of the Δ*K* measurements, which are the cornerstone of DIP, is directly proportional to the length of internal branches, meaning that DIP gains power to differentiate between alternative hypotheses as branches are lengthened. Finally, there is a disparity in the accuracy of topology classification for loci introgressed *P3⇒P2* versus the opposite direction (Zheng and Janke 2018). This disparity stems from the fact that the internal branch on *P2⇒P3* introgression gene trees is shorter than the same branch on *P3⇒P2* introgression gene trees, making for fewer diagnostic synapomorphies by which to infer the introgression topology. This disparity is most pronounced under conditions in which phylogenetically informative synapomorphies are scarce (i.e., short branch lengths). Moreover, the specific disparity between genes introgressed in each direction has an important consequence for simulation analyses, the short internal branch on *P2⇒P3* introgression gene trees results in a higher rate of ILS for these loci compared with other categories of loci, meaning that ILS obscures the introgression history of these loci at a higher rate than loci introgressed in the opposite direction. This disparity is especially problematic for DIP because it is likely to introduce a directional bias, favoring inference of *P3⇒P2* introgression.

For the above reasons, we performed parameter scans to explore the influence of branch lengths and timing of introgression. We found that 2×DIP performs as expected when the classification step is bypassed in omniscient mode (fig. 6*A*, *D*, and *G*) but bias at short branch lengths arises when introgressed and nonintrogressed loci must be classified directly based on the data (fig. 6*B*, *E*, and *H*). When working with empirical data sets, omniscience about origins and the effects of introgression versus ILS on individual loci is not possible. As such, classification error may be unavoidable, so we sought to develop a strategy to correct for bias that arises from it, leading to the development of 3×DIP. A benefit of 3×DIP is that it is applicable under the conditions in which bias is most pronounced. Following the logic of the *D-*statistic (Green et al. 2010), 3×DIP is based on the expectation that ILS is equally likely to produce the two topologies that conflict with the species tree: (*P1*(*P2*, *P3*)) and (*P2*(*P1*, *P3*)). Therefore, under the assumption that there has been no introgression between *P3* and *P1*, the number of ALT loci, which are defined by having the (*P2*(*P1*, *P3*)) topology, provides an estimate for the number of identified loci displaying the introgressed topology that were actually the result of ILS. Accordingly, 3×DIP applies a correction for ILS that is proportional to the frequency of these ALT loci. We found that 3×DIP reduces directional bias at short branch lengths (fig. 6*C*, *F*, and *I* and fig. 7) and does not provide false positive results in the complete absence of introgression (supplementary fig. S5, Supplementary Material online). These results indicate that 3×DIP is a step toward overcoming directional bias; however, bias persisted for the shortest branch-length simulations, meaning that there are biological scenarios in which 3×DIP is not free from bias. Further, under situations in which introgression occurs immediately following speciation, we observed cases in which 2×DIP exhibited less bias than 3×DIP (supplementary fig. S4*G*, Supplementary Material online).

The basic premise of 3×DIP is that the number of ALT loci serves as a proxy for the number of loci that have a true history of speciation but display an introgression topology due to ILS. This assumption appears valid in a scenario with ILS but not introgression, as indicated by the ability of 3×DIP to eliminate bias under these simulated conditions (supplementary fig. S5, Supplementary Material online). However, 3×DIP does not account for the fact that ILS occurs not only for loci with a speciation history but also loci with an introgression history. In other words, some of the loci that exhibit the ALT topology will have a true history of introgression, making these loci an imperfect proxy for the number of loci with a speciation history affected by ILS. This can cause undesired behavior of 3×DIP in situations in which most or all of the ALT topologies stem from loci with a history of *P2⇒P3* introgression. Therefore, we suggest that there is a benefit to applying all three variations of DIP to provide the most comprehensive view of introgression directionality.

Fully overcoming bias introduced into introgression analyses by classification error represents a future goal for the field. With current implementations of DIP, inferences of introgression in the *P3⇒P2* direction should be viewed with caution, especially in taxa with very recent divergence times or when introgression occurred very shortly after a speciation event. On the other hand, it can be viewed as a conservative test for *P2⇒P3* introgression, so identification of introgression in that direction can be interpreted as a much more confident prediction. As suggested above, further progress in this area may come through more complex models that explicitly include ILS that occurs at introgressed loci (Hibbins and Hahn 2019), rather than solely at nonintrogressed loci.

A related challenge to DIP analyses is associated with the question of how to partition the genome. Arbitrarily breaking chromosomes into loci of a fixed size may be problematic because the resulting “loci” may either be composed of multiple haplotype blocks with different genealogies due to intralocus recombination or, conversely, an individual haplotype block may contain multiple partitioned “loci,” resulting in pseudoreplication as it will be sampled numerous times by DIP. Our simulations of introgression and recombination revealed that these issues do not introduce a directional bias but do dramatically increase the variance of DIP when the size of true haplotype blocks is much larger than the window size used by DIP. One potential strategy for mitigating this challenge would be to incorporate methods that explicitly infer recombination breakpoints (e.g., the four-game test; Hudson and Kaplan 1985) into the window-definition phase of DIP.

There are also unexplored factors that should be considered when implementing DIP because our simulations were run under simplifying assumptions such as random mating, constant population size, and a single bout of instantaneous introgression solely between *P3* and *P2*. Violation of these assumptions in natural populations (Eriksson and Manica 2012; Prüfer et al. 2014; Kuhlwilm et al. 2016; Slon et al. 2018) may introduce additional sources of bias, Our simulation strategies also do not fully capture rate heterogeneity across the genome, branch-specific variation in effective population size/mutation rate, technical biases caused by read-mapping, and introgression from unsampled taxa (i.e., “ghost lineages”). These factors should be investigated in future studies with more complex simulation scenarios.

### DIP Performance on Empirical Data

We chose hominin introgression as a test case because it is one of the most famous and best-studied examples of introgression. An additional benefit is that the sampling in the group is dense; several modern human samples as well as samples from ancient Neanderthal and Denisovan tissues are available. A benefit of this dense taxon-sampling is that previous studies have been able to apply five-taxon statistics to polarize introgression, leading to the conclusion that “all or almost all of the gene flow detected was from Neandertals into modern humans” (Green et al. 2010). However, more recent analyses of additional archaic samples from different parts of the hominin geographical range also indicated introgression in the opposite direction (Kuhlwilm et al. 2016) as well as mating between Neanderthals and Denisovans (Slon et al. 2018).

An additional benefit of dense hominin taxon-sampling is that the phylogenetic placement of samples allows us to analyze the same introgression event with four-taxon statistics from two different angles. We devised a TSS in which Neanderthal and a modern human acted as *P3* and *P2*, respectively (TSS1, fig. 8*A*) as well as one in which the roles were reversed (TSS2, fig. 8*F*). Importantly, these TSSs allowed us to evaluate whether the directional bias described above was strong enough to outweigh the true signature from introgression. DIP returned contradictory results for TSS1 and TSS2. In both cases, 2×DIP and 3×DIP favored *P3⇒P2* introgression, despite the identity of *P3* and *P2* being reversed in the two cases. The fact that both analyses sided with the directional bias we documented above, suggests that bias may be outweighing the introgression signature. This is consistent with the observation that hominin divergence is both lower and more heterogenous than our simulated branch lengths (supplementary fig. S6, Supplementary Material online), suggesting that biasing factors are strong enough to bias even 3×DIP*.* It is worth noting, however, that the magnitude of ΔΔ*K* from TSS1 is higher than that from TSS2 and the variance of ΔΔΔ*K* is much larger for TSS2 than for TSS1, meaning the signal favoring Neanderthal⇒French introgression (the expected direction) is stronger and less noisy than the signal in the opposite direction.

Our general takeaway from analysis of hominin data is that, like all introgression analysis tools, there are limits to the conditions under which DIP can be reliably applied. Although 3×DIP may represent a step in the right direction, in the case of hominin introgression, the level of ILS swamps out the signal of introgression. We suggest that incorporating an alternative means of identifying introgressed loci, such as *f*_d_ (Durand et al. 2011; Martin et al. 2015), may yield more reliable results when ILS is prevalent, representing an area of future work. For the time being, DIP will be most reliable in cases of introgression that occurred at more ancient time scales (Forsythe et al. 2018; Dasmahapatra et al. 2012; Fontaine et al. 2015).

## Materials and Methods

### Resource Availability

URLs for downloading previously published data are provided in place in the following sections. Scripts for reproducing the analyses in this study are available at:https://github.com/EvanForsythe/DIP. In addition included are *R* scripts for performing DIP on genomic data. All scripts are callable from the command line. Users have the choice of inputting either whole-chromosome alignments, which will be divided into single-window (i.e., locus) alignments in preparation for DIP. Alternatively, DIP takes single-locus alignments, bypassing the window partitioning step. DIP outputs descriptive statistics and PDF figures similar to figure 8.

### Simulations of Sequence Evolution

We generated whole-genome alignments in which introgression has occurred in some (but not all) loci, and in which donor and recipient taxa for each introgressed locus are known. To accomplish this, we simulated sequence evolution of loci 5,000 nucleotides in length in a four-taxon system (three in-group taxa, *P1*, *P2*, and *P3* and an outgroup, *O*) (fig. 1). All simulations were performed with *ms* (Hudson 2002) and *seq-gen* (Rambaut and Grassly 1997) implemented in *R* v3.5.0 with *phyclust* v0.1-22 (Chen 2011) similar to Martin et al. (2015). *Ms* was used to generate a coalescence tree, which was passed to *seq-gen* in order to generate a sequence alignment. A portion of the loci were simulated to have evolved along a path of simple speciation. In the absence of ILS, the gene trees for these loci should match the speciation history, ((*P1, P2*)*P3*)*O*) (fig. 1*A*). These loci, denoted as species topology loci, were simulated with the following *R* commands:

ret.msSP<-ms(nsam = 4, nreps = 1, opts = “-T -t 50 -I 4 1 1 1 1 -ej 4 2 1 -ej 8 3 1 -ej 12 4 1 -r 5 5000”)

seqsSP<-seqgen(opts = “-mHKY -l5000 -s 0.01”, newick.tree = ret.msSP[3])

In the above *ms* call, the -T argument directs *ms* to output gene trees, one of which is used as input for *seq-gen*. The -t argument sets the *theta* value used by *ms*, which was held constant across all simulations. The arguments -I 4 1 1 1 1 indicate that four populations were simulated with one individual sampled from each, which was also held constant across all simulations. Each -ej command represents a speciation event (in a forward-time context), the first number following the -ej flag being the timing of the event and the two following numbers being the two daughter populations arising from the speciation. The -r argument indicates the rate of recombination and the final number indicates the length of the segments being simulated by *ms*. However, for this simulation strategy, we only input one tree into seq-gen, essentially simulating nonrecombining loci (however, see below for our explicit treatment of recombination).

Loci with instantaneous unidirectional introgression occurring between *P2* and *P3* were also simulated. Introgression trees (transferred in either direction) will have the topology, (*P3, P2*)*P1*)*O*), and thus differ from the species tree. The direction of introgression for an individual locus was indicated by “donor taxon” and “recipient taxon” as in the following *R* command:

ret.msINT <- ms(nsam = 4, nreps = 1, opts= “-T -t 50 -I 4 1 1 1 1 -ej 4 2 1 -ej 8 3 1 -ej 12 4 1 -es 2 <recipient taxon> 0.4 -ej 2 5 <donor taxon> -r 5 5000”)

seqsINT<-seqgen(opts = “-mHKY -l5000 -s 0.01”, newick.tree = ret.msINT[3])

We replicated the above commands for species and introgressed topology loci to create data sets representing simulated whole-genome alignments composed of a total of 5,000 loci (supplementary fig. S1, Supplementary Material online). The argument in the above command that specify introgression are the -es argument and the final -ej command. We define the proportion of all loci in the genome resulting from simulated introgression in either direction as *pINT* and the proportion of introgressed genes that were transferred in the *P3⇒P2* direction as *p(P3⇒P2).* Because a single locus can only be transferred in one direction or the other, the proportion of loci transferred in the *P2⇒P3* direction, *p(P2⇒P3)*, is 1−*p(P3⇒P2).* Whole-genome alignments with known values of *pINT* and *p(P3⇒P2)* were used to test the performance of DIP. We performed parameter scans by simulating genome alignments with varying combinations of *pINT* and *p(P3⇒P2)* (see supplementary fig. S1, Supplementary Material online).

Recognizing that the above simulation strategy does not realistically model recombination, we also employed an alternative simulation strategy in which we simulate whole chromosomes (rather than individual loci) while allowing for varying levels of recombination. Introgression in the presence of recombination was simulated with the following *ms* command in *R*.

ms(nsam = 4, nreps = 1, opts = T -t 50 -I 4 1 1 1 1 -ej 4 2 1 -ej 8 3 1 -ej 12 4 1 -es 1 <recipient taxon> <pINT> -ej 1 5 <donor taxon> -r <recombination rate> 12500000)

The output files from the above *ms* command (run twice in cases of bidirectional introgression—once for each direction of introgression) were combined into a single file, which was input to *seq-gen* in order to generate a whole-chromosome alignment*. Seq-gen* was called from the command line with the following command:

seq-gen -mHKY -l 25000000 -s 0.01 -p <number of haplotype blocks from ms> < <ms_output_file> > <seqgen output file name> 2> <file name to store haplotype block positions>

Whole-chromosome alignments were replicated five times for each parameter value and DIP analyses were performed with the 5,000-bp partitioning approach applied elsewhere in this article.

The default branch length parameters used for figures 3 and 5 are *T*_INT_=1, *T*_α_=4, *T*_β_=8, and *T*_γ_=12 measured in coalescent units of 4 *N* generations (see fig. 1). To explore the effects of divergence times, we multiplied all branch length parameters by a range of different SF values. For example, SF = 0.1 results in the following node depths: *T*_INT_=0.1, *T*_α_*=*0.4*, T*_β_=0.8, and *T*_γ_=1.2.

As an additional means of exploring the effects of speciation and introgression timing, we also varied the timing of introgression in proportion to the most recent speciation even (relative introgression time). The timing of introgression was set relative to the *T*_α_ speciation time. For example, under default SF described in the previous paragraph with *T*_α_*=*4, a relative introgression time of 0.8 translates to *T*_INT_=3.2. For parameter scans involving branch lengths, we generated point estimates of ΔΔ*K* and ΔΔΔ*K* from five replicate genomes for each condition.

### Classification of Introgressed and Nonintrogressed Loci

The first step in all versions of DIP is sorting loci to distinguish the loci that were introgressed from those that follow the species branching order (i.e., classification). Using simulated data affords us omniscience at this step (i.e., we know whether each locus was originally simulated as introgressed or not). However, unless specifically stated, we did not make use of the known history of simulated loci. Instead, DIP infers the introgression status of loci based on the topology of a neighbor-joining gene tree inferred for each locus using *Ape* v5.2 (Paradis et al. 2004). Loci displaying the ((*P1, P2*)*P3*)*O*) topology are marked as nonintrogressed loci. Loci displaying the ((*P2, P3*)*P1*)*O*) topology (introgressed topology) are designated as introgressed loci. Any loci displaying the alternative topology, ((*P1, P3*)*P2*)*O*), which are not produced by speciation or introgression, are omitted from 1×DIP and 2×DIP but used by 3×DIP to calculate a correction factor (see below).

### Inferring Introgression Directionality with 1×DIP

We calculated the pairwise divergences, *K*_23_, *K*_12_, and *K*_13_ (as indicated in fig. 1*A*) for each locus using the *dist.dna* command from the *Ape* package with default settings. Pairwise divergences, *K*_23_, *K*_12_, and *K*_13_ are named for the taxa involved in the distance calculation. For example, *K*_23_ measures the divergence of *P2* and *P3* (see fig. 1). Δ*K*_23_, Δ*K*_12_, and Δ*K*_13_ were calculated based on difference in mean *K* values between SP and introgression loci as shown in equations (1–3). To test for significance, bootstrapped distributions were obtained by resampling (with replacement) loci from the genome to achieve genome alignments equal in number of loci to the original genome alignment. 1,000 such replicates were performed, recalculating Δ*K*_23_, Δ*K*_12_, and Δ*K*_13_ for each replicate. *P* values for the significance of Δ*K* values were calculated as the proportion of replicates for which Δ*K* ≤ 0. For the parameter scan of 1×DIP (fig. 3*D*), inference of a significant directional profile required that all three measures, Δ*K*_23_, Δ*K*_12_, and Δ*K*_13_, adhere to their expected profile with a significant (*P *<* *0.05) *P* value for each (with the exception of cases in which the expectation is Δ*K *=* *0).

### Inferring Introgression Directionality with 2×DIP and 3×DIP

ΔΔ*K* was calculated from Δ*K*_12_ and Δ*K*_13_ as described in equation (4). The bootstrap resampling scheme described in the previous paragraph was used to assess the significance of 2×DIP. ΔΔ*K* was calculated for each replicate and *P* values were obtained from the proportion of replicates for which ΔΔ*K* overlapped zero (multiplied by two for a two-sided test). Like 2×DIP, 3×DIP makes use of ΔΔ*K* to indicate the directionality of introgression. However, 3×DIP also introduces ΔΔ*K*_ALT_, which is calculated according to equation (5). ΔΔΔ*K* is obtained from the difference between ΔΔ*K* and ΔΔ*K*_ALT_ (see eq. 6).

The rationale for the 3×DIP correction is that the observed value of ΔΔ*K* may be viewed as a weighted average of: 1) a corrected value (ΔΔΔ*K*) that is based only on the loci that truly experienced a history of introgression and 2) a spurious signal (ΔΔ*K*_ILS_) arising from the unknown number of loci that exhibit an introgression topology that is actually the result of ILS (*N*_ILS_).

(7) $$\Delta \Delta K=\left(\frac{N_{\mathrm{INT}}\;-\;N_{\mathrm{ILS}}}{N_{\mathrm{INT}}}\right)\Delta \Delta \Delta K+\left(\frac{N_{\mathrm{ILS}}}{N_{\mathrm{INT}}}\right)\Delta \Delta K_{\mathrm{ILS}}$$

Based on the expected symmetry of ILS, we can use ΔΔ*K*_ALT_ and *N*_ALT_ as estimates of ΔΔ*K*_ILS_ and *N*_ILS_, respectively.

(8) $$\Delta \Delta K=\left(\frac{N_{\mathrm{INT}}\;-\;N_{\mathrm{ALT}}}{N_{\mathrm{INT}}}\right)\Delta \Delta \Delta K+\left(\frac{N_{\mathrm{ALT}}}{N_{\mathrm{INT}}}\right)\Delta \Delta K_{\mathrm{ALT}}$$

Solving equation (8) for ΔΔΔ*K* yields equation (6) (see Results). This approach is based on substantial simplifying assumptions. For example, it does not account for the misidentification of loci that have a true history of introgression but exhibit the species or ALT topology because of ILS (see Discussion). As for ΔΔ*K* above, significance of ΔΔΔ*K* is obtained from resampled whole-genome alignments.

### Hominin Data Analysis

To generate whole-chromosome alignments from the hominin data set for DIP, Chromosome I sequencing data for two Neanderthal, one Denisovan, and two modern human samples from Prüfer et al. (2014) were downloaded from http://cdna.eva.mpg.de/neandertal/ (last accessed March 25, 2020) as VCF files. The human reference genome (hg19) (International Human Genome Sequencing Consortium 2001), which was originally used for read-mapping during the creation of VCF files, was obtained from http://hgdownload.cse.ucsc.edu/goldenPath/hg19/ (last accessed March 25, 2020).

Structural variation (indel) information was trimmed from VCF files, using *VCFtools v. 0.1.13* (Danecek et al. 2011) and *Tabix* (Li et al. 2009) with the following commands:

vcftools –gzvcf Chrom1_with_indels.vcf.gz –remove-indels –recode –recode-INFO-all –out Chrom1_SNPs_only.vcf

bgzip Chrom1_SNPs_only.vcf

tabix -p vcf Chrom1_SNPs_only.vcf.gz

Whole-chromosome consensus sequence was extracted from VCF files using *BCFtools v1.6* (Li et al. 2009) with the command below. For heterozygous sites, by default *bcftools consensus* applies the alternative variant (i.e., the variant that does not match the reference genome) to the consensus sequence for the given sample (see https://samtools.github.io/bcftools/bcftools.html, last accessed March 25, 2020). It should be noted that heterozygosity information may be lost at this step, which was necessary to match the format of the phylogenetic data generated in our simulations.

cat hg19_chrom1.fa | bcftools consensus Chrom1_SNPs_only.vcf.gz > Chrom1_cons.fa

We used the reference chimpanzee genome (PanTro5) (The Chimpanzee Sequencing Consortium 2005) as an outgroup. We downloaded a MAF alignment of chromosome one from PanTro5 and hg19 from: http://hgdownload.cse.ucsc.edu/goldenpath/hg19/vsPanTro5/axtNet/ (last accessed March25, 2020). We converted this file to FASTA format using Galaxy tools (Afgan et al. 2018) available at https://usegalaxy.org/ (last accessed March 25, 2020). Finally, the consensus sequence from each hominin sample and chimpanzee was concatenated into a whole-chromosome multiple sequence alignment in FASTA format. This five-taxon alignment was pruned to contain four taxa according to each TSS (see fig. 8) and then divided into single-locus alignments 5,000 bp in length, which were used as input to DIP. 

## Supplementary Material

Supplementary data are available at *Genome Biology and Evolution* online.

## Supplementary Material

evaa053_Supplementary_Data
