# Supplementary material for: Divergence-Based Introgression Polarization
Source: Genome Biol Evol. 2020 Mar 27;12(4):463–78. doi: 10.1093/gbe/evaa053 (PMC7197497; doi:10.1093/gbe/evaa053)
Supplement: evaa053_Supplementary_Data [file evaa053_supplementary_data.docx]

**SUPPLEMENTAL INFORMATION**

**Fig. S1. Schematic of the workflow used to simulate introgression across a genome and perform *DIP*. (1)** Each locus is evolved along the species tree or along a path of introgression and used to generate a 5000-bp alignment using *ms* and *seq-gen* similar to (Martin et al. 2015). **(2)** Step 1 was repeated to yield a full genome of *n=*5000 loci in which *n* x *pINT* loci were introgressed and the remaining loci evolved along the species tree. For example, a genome in which half of all genes were not transferred while the other half were transferred *P3⇒P2* would be generated with: *n=5000, pINT* = 0.5, *p(P3⇒P2)* = 1.0. **(3)** Different steps in the *DIP* pipeline are performed on the simulated genome. **(4)** Steps 1-3 are repeated for each combination of *pINT* and *p(P3*⇒*P2)*. Each pixel in a parameter scan graph represents one or more runs of Steps 1-3.

**Fig. S2. 2xDIP analyses of introgression simulated with recombination.** Genomes were simulated using an alternative simulation strategy that includes recombination (see Methods). Three different recombination rates were used, r=7 (A and D), r=70 (B and E), and r=700 (C and F), resulting in haplotypes of different sizes. Five replicate genomes were simulated for each value of *p(P3⇒P2)*. To provide a understanding of the haplotype blocks with diagnostic synapomorphies that exist at different levels of recombination, the number of instances in which neighboring blocks exhibit different topologies (i.e. topology changes) were summed (D-F). Note that number of topology changes differs from haplotype blocks because some SNPs occurred along branches that don’t define the topology (i.e. not all SNPs represent diagnostic synapomorphies).

**Fig. S3. Gene tree topologies inferred from simulated genomes.** Gene tree counts for genomes simulated with different branch lengths (x-axes) and *p(P3⇒P2)* values of 0.6 (**A**), 0.5 (**B**), and 0.4 (**C**). Each point represents the number of trees displaying a given topology from a replicate genome. ((*P1,P2*)*,P3*)*, orange;* ((*P2,P3*)*,P1*)*, green;* ((*P1,P3*)*,P2*)*, purple.* These same simulated genomes were analyzed in Fig. 6.

**Fig. S4. Simulations of relative introgression timing.** (A-F) Genomes were simulated using two different scaling factors, SF=1 (A-C) and SF=0.1 (D-F) to determine the timing of speciation events. While the timing of all speciation events adhere to the given scaling factor, the timing of the introgression event (*T_INT_*) was set as a fraction of the timing of the most recent speciation event (*T_α_*) (i.e. relative introgression time). High relative introgression time indicates introgression occurred directly following speciation whereas low relative introgression time indicates introgression occurred a long duration of time following speciation. Three different types of asymmetrical/symmetrical bidirectional introgression were simulated for each relative introgression time, *p(P3⇒P2)*= 0.4 (purple), *p(P3⇒P2)* = 0.5 (orange), and *p(P3⇒P2)* = 0.6 (green). Omniscient *2*×*DIP* (A and D), non-omniscient *2*×*DIP* (B and E), and *3*×*DIP* (C and F) were performed on all genomes. (G-H) *ΔΔK* (blue) and *ΔΔΔK* (dark red) measurements for genomes simulated with *p(P3⇒P2)* = 0.5 and different scaling factors. Five replicate genomes were simulated for each parameter value.

**Fig. S5. *DIP* analysis of a genome with incomplete lineage sorting but no introgression.** A genome alignment was simulated with *pINT* set to zero using the scaling factor 0.1 (see Fig. 1 and Fig. 6). Therefore, all loci with topologies that conflict with species tree are the result of ILS and not introgression (**A**) The topologies of neighbor joining trees inferred from 5000 simulated loci. ((*P1,P2*)*,P3*)*, green;* ((*P2,P3*)*,P1*)*, orange;* ((*P1,P3*)*,P2*)*, purple.* (**B-D**) 1×*DIP* (**B**), 2×*DIP* (**C**) and 3×*DIP* (**D**) analysis of the genome alignment. ­

**Fig. S6. Sequence divergence measures from simulated and Hominin data.** Violin plot showing distributions of pairwise divergence values for inferred loci displaying the species (SP) and introgressed (INT) topology (see Fig. 1 and 2). Both simulated datasets were simulated with *pINT*=0.5 and *p(P3⇒P2)*=0.5.
